# Network-wide aberrancies in neuronal activity during working memory in a large cohort of patients with mood disorders: associations with cognitive impairment and functional disability

**DOI:** 10.1038/s41380-025-03078-x

**Published:** 2025-06-17

**Authors:** Viktoria Damgaard, Johanna Mariegaard Schandorff, Julian Macoveanu, Anjali Sankar, Jeff Zarp, Patrick M. Fisher, Martin Balslev Jørgensen, Lars Vedel Kessing, Gitte Moos Knudsen, Vibe G. Frokjaer, Kamilla Woznica Miskowiak

**Affiliations:** 1 Neurocognition and Emotion Across Disorders of the Brain (NEAD) Centre, Psychiatric Centre Copenhagen, Frederiksberg Hospital, Frederiksberg, Denmark; 2 Department of Psychology, University of Copenhagen, Copenhagen, Denmark; 3 Neurobiology Research Unit, Rigshospitalet, Copenhagen, Denmark; 4 Department of Drug Design and Pharmacology, University of Copenhagen, Copenhagen, Denmark; 5 Department of Clinical Medicine, University of Copenhagen, Copenhagen, Denmark; 6 Copenhagen Affective Disorder Research Centre (CADIC), Psychiatric Centre Copenhagen, Frederiksberg Hospital, Frederiksberg, Denmark; 7 Mental Health Centre Copenhagen, Copenhagen University Hospital – Mental Health Services Copenhagen, Copenhagen, Denmark

**Keywords:** Bipolar disorder, Depression, Psychology, Neuroscience

## Abstract

Individuals with mood disorders present with cognitive impairment and functional disability, and small-scale studies underline aberrant cognitive control and default mode network activity as potential neuronal correlates underlying these deficiencies. The objectives of this large-scale, cross-sectional functional magnetic resonance imaging (fMRI) study were (I) to investigate the replicability of cognitive control network (CCN) hypo-activity and default mode network (DMN) hyper-activity in patients with mood disorders, and (II) to explore brain activity related to cognition and daily functioning across patients and controls. We pooled data from three studies conducted at the same study site, which resulted in a sample of 213 fully or partially remitted patients with mood disorders (189 with bipolar disorder, 24 with major depressive disorder) and 60 healthy controls (HC). All participants underwent fMRI, during which they performed a verbal working memory N-back task, as well as comprehensive neurocognitive testing and assessment of daily functioning. Patients showed task-related hypo-activity within the left dorsolateral prefrontal cortex as well as frontal and parietal nodes of the CCN, which correlated with poorer outside-scanner cognitive performance. Within the DMN, patients showed hyper-activity in the frontal medial cortex compared to HC. Cognitive performance was positively associated with task activity within the right middle frontal gyrus (*p* = 0.0005), located in the CCN, whereas daily functioning was negatively associated with activity within the cingulate gyrus, a key hub in the DMN (*p* = 0.007). In the largest study of its kind, we identified CCN and DMN abnormalities in mood disorders and associations with cognition and functioning. The findings highlight plausible neurocircuitry targets for enhancing cognitive and functional recovery in mood disorders.

## Introduction

Cognitive impairment and associated functional disability are prevalent in patients with mood disorders like major depressive disorder (MDD) and bipolar disorder (BD), also during euthymia [1–3]. These impairments are associated with worse clinical outcomes, including risk of psychiatric hospitalization [4] and poorer psychopharmacological treatment efficacy [5]. Currently, there are no robust and enduring treatment strategies targeting cognition and daily functioning in mood disorders [6]. While meta-analytic findings suggest potential beneficial effects of cognitive remediation therapy on cognitive performance in mood disorders, these effects are solely mild to moderate and do not seem to transfer sufficiently to improved daily life functioning [7, 8]. This lack of effective treatment strategies is partly due to our limited understanding of the precise neuronal underpinnings of these cognitive and functional abilities and thereby which neurocircuitries to directly target to enhance cognitive performance and daily functioning.

Functional magnetic resonance imaging (fMRI) studies in patients with mood disorders have demonstrated task-related aberrant activation in the frontoparietal cognitive control network (CCN) as well as the default mode network (DMN) during cognitive performance [9]. This has mainly been investigated using working memory (WM) N-back tasks. Specifically, studies have reported increased WM-related activity in the DMN in mood disorders compared to controls [10, 11], while findings in the CCN are less uniform. Here, the direction of aberrant CCN activity seems to vary with cognitive performance. In particular, WM fMRI studies comparing cognitively impaired vs. cognitively intact patients have demonstrated neuronal hypo-activation within the PFC and frontal and parietal nodes of the CCN, in addition to increased activity within the default mode network (DMN), in cognitively impaired patients only [12, 13]. Conversely, other studies have shown WM-related hyper-activity in the CCN coupled with intact cognitive performance in mood disorders [14, 15], which may reflect reduced neuronal efficiency in the CCN as patients need to recruit additional cortical resources to maintain their performance [9].

Despite the importance of functional recovery in patients with mood disorders, there appear to be only few studies investigating brain activation patterns associated with daily life functioning. Specifically, one small fMRI study in 10 euthymic patients with BD observed a positive association between psychosocial functioning and activity within the left anterior cingulate and lateral PFC during a verbal fluency task [16]. Another more recent study of 31 euthymic patients with BD demonstrated significant positive correlations between daily functioning and WM-related brain activity within the dorsolateral PFC, inferior frontal gyrus, and temporal-occipital areas [17]. This has also been observed across patients with both BD and MDD, where lower WM-related activity within the dorsolateral PFC was associated with poorer daily functioning [18]. Taken together, this suggests that hypo-activity within the PFC and CCN may also underlie daily life functional impairments, in addition to cognitive dysfunction, in individuals with mood disorders during euthymic periods. Nevertheless, current fMRI studies in mood disorders are generally limited by small sample sizes (typically, *n* < 50 [9]). Consequently, investigating the replicability of these findings in a larger sample is highly warranted. Moreover, only few studies have assessed the relationship between neuronal activity and comprehensive outside-scanner cognitive performance and daily life functioning, which impedes key insights into the *functional* implications of neurocircuitry activity [19].

The objective of this large-scale, cross-sectional functional magnetic resonance imaging (fMRI) study of patients with mood disorders was twofold. Firstly, we wanted to assess replicability of hypothesized WM-related neuronal activity differences between patients and controls in the largest study of its kind to date, and to examine if brain regions showing significant group differences were related to cognitive impairment and functional disability in patients. For this first aim, we hypothesized that (i) patients showed aberrant hypo-activity within the PFC and CCN coupled with increased activity in the DMN compared to controls, and that (ii) this aberrant network-wide neuronal activity was associated with both cognitive impairment and poor functioning. Secondly, using a whole brain exploratory approach, we wanted to examine the relation between task-related neurocircuitry activity and comprehensive outside-scanner measures of cognition and daily functioning across patients with mood disorders and controls.

## Methods

### Participants and procedure

We pooled baseline neurocognitive and fMRI data from patients with mood disorders and healthy controls (HC) from three prior studies conducted at the same research university hospital site in Copenhagen, Denmark: the Bipolar Illness Onset study [20], and the Prefrontal Target Engagement as a biomarker model for Cognitive improvement (PRETEC) studies [21, 22], respectively. This resulted in a total sample of 213 patients with mood disorders (189 BD/24 MDD) and 60 HC. Pooling data from these prior studies was appropriate given the overlapping inclusion criteria, as well as the equal administration of neurocognitive tests, ratings of daily functioning and mood symptoms, and fMRI paradigm.

All patients had an ICD-10 diagnosis of MDD or BD, which was verified with the Schedules for Clinical Assessment in Neuropsychiatry (SCAN) [23] by trained PhD students or Postdoctoral researchers, were in full or partial remission at the time of inclusion (defined as ≤14 on the Hamilton Depression Rating Scale – 17 items version (HDRS-17) [24] and Young Mania Rating Scale (YMRS) [25]), were between 18 and 65 years of age, and fluent in Danish. Patients from one of the PRETEC studies were also free of any major somatic disorder (for details, see [26]). Healthy participants had no personal or first-degree relative history of psychiatric disorder. Details for recruitment procedures and exclusion criteria are available from ClinicalTrials.gov (BIO: NCT02888262; PRETEC-ABC: NCT03295305; PRETEC-EPO: NCT03315897). On the day of assessment, participants underwent neurocognitive testing, ratings of daily functioning and mood symptoms, and, within 0–3 days, an fMRI scan. All studies have been approved by the Danish Research Ethics Committee for the Capital Region of Denmark (BIO: H-72014007; PRETEC-EPO: H-16043370; PRETEC-ABC: H-16043480) and The Danish Data Protection Agency Capital Region of Denmark (BIO: RHP-2015-023; PRETEC: 2012-58-0004). Written informed consent was obtained for all participants prior to participation in their respective studies.

### Measures

#### Demographics, IQ, and mood symptoms

We collected demographic characteristics (age, sex, and years of education) as well as an estimate of a verbal intelligence quotient (IQ) using the Danish Adult Reading Task (DART) [27]. Participants’ subsyndromal mood symptoms were further assessed with the HDRS-17 [24] and YMRS [25] scales, respectively.

#### Neurocognitive and daily functioning assessment

Cognition was assessed using a comprehensive neuropsychological test battery outside the scanner, including the Rey Auditory Verbal Learning Test (RAVLT) [28], Trail Making Test Part A and B (TMT-A, TMT-B) [29], The Repeatable Battery for the Assessment of Neuropsychological Status (RBANS) Coding & Digit Span [30], Wechsler Adult Intelligence Scale (WAIS)-III Letter-Number Sequencing [31], Verbal Fluency letters 'S' and 'D' [32], Screening for Cognitive Impairment in Psychiatry (SCIP-III) [33], and the Rapid Visual Processing (RVP), Spatial Working Memory (SWM), and One Touch Stockings of Cambridge (OTS) from CANTAB (Cambridge Cognition Ltd.).

Daily functioning was assessed with the observer-rated, interview-based Functioning Assessment Short Test (FAST) scale, which has shown strong psychometric properties [34]. The scale evaluates six domains of functioning: autonomy, occupational functioning, cognitive functioning, financial issues, interpersonal relationships, and leisure time. A higher FAST total score reflects higher functional disability.

#### Verbal N-back fMRI task

The fMRI paradigm applied was a verbal letter-variant N-back task displayed on a screen, which participants viewed through an angled mirror in the scanner. Participants were instructed to respond with a right index finger button press on a response pad when a letter stimulus matched the one occurring *N* (1, 2, or 3) steps back in the letter sequence. The task thus had three levels of cognitive load: 1-back, 2-back, and 3-back. Additionally, participants completed a sensorimotor control condition (0-back) which required them to respond when seeing the letter X. Each N-back task load was presented in four blocks in a fixed pseudorandom order after a instruction screen for 12 s. Each block contained 10 letters, including 3 target stimuli, shown for 0.5 s each with a fixed interstimulus interval of 1.5 s. Blocks were separated by a 5 s fixation cross. The total duration of the task was 9 minutes and 52 seconds. Response accuracy and time latency were recorded with ePrime 2.0 (Psychological Software Tools, Pittsburgh, PA, USA). However, due to technical issues during the fMRI sessions, these behavioral data were corrupted and thus not available for analysis.

### Statistical analysis of behavioral data

Data normality distributions were explored using Shapiro-Wilk test of normality and evaluation of histograms for each variable of interest. Independent samples *t*-tests, Pearson’s Chi-square (χ^2^), and nonparametric Mann-Whitney *U* tests were conducted to assess group differences in demographic variables, mood symptoms, cognition, and daily functioning.

Neuropsychological test raw scores were *z*-transformed based on the means and standard deviations (SD) from the HC sample as well as available norms for the SCIP-III [35] and CANTAB OTS [36], as none of the healthy participants had performed these two tests. The *z*-transformed scores were then averaged within five domain composite scores (‘Attention’, ‘Psychomotor speed’, ‘Working memory’, ‘Verbal fluency and executive function’, and ‘Verbal learning and memory’; see Supplementary Table S1 for the specific tests grouped within each of these domain-specific composites). A ‘Global cognition composite’ score was further computed as an average of these five cognitive domain scores. We used R studio 4.2.2 (version 2022.12.0 + 353) for all statistical analyses with a set significance level threshold at *p* ≤ 0.05 (two-tailed).

### Functional MRI analysis

#### fMRI data acquisition

Functional MRI data were acquired at the Copenhagen University Hospital, Rigshospitalet using a 3 Tesla Siemens Prisma scanner and a 64-channel head-coil. During the verbal N-back task, blood oxygen level dependent (BOLD) fMRI was acquired using a T2*-weighted gradient echo spiral echo-planar (EPI) sequence with an echo time (TE) of 30 ms, repetition time (TR) of 2 s, and flip angle of 90°. A total of 300 volumes were obtained, each containing 32 slices with a thickness of 3 mm with 25% gaps in-between, and a field of view (FOV) of 230 × 230 mm using a 64 × 64 grid. BOLD images were registered to T1-weighted structural images (TR = 1900 ms; TE = 2.58 ms; flip angle = 9°; distance factor = 50%; FOV = 230 × 230 mm; slice thickness = 0.9 mm). We also acquired a standard B0 field map sequence with the same FOV and resolution as the fMRI sequence (TR = 400 ms; TE = 7.38 ms; flip angle = 60°) used for geometric distortions correction of the BOLD images. Image quality was ensured by visual inspection of all individual images.

#### fMRI pre-processing and first-level analysis

Functional MRI data processing was performed with the FMRI Expert Analysis Tool (FEAT; version 6.0.5), in FSL (FSL; www.fmrib.ox.ac.uk/fsl). Pre-processing included image realignment, non-brain removal, spatial normalization to a Montreal Neurological Institute (MNI) template, and spatial smoothing with a 5 mm full-width-half-maximum Gaussian kernel. A high-pass temporal filtering cutoff of 100 s was applied. Correction for geometric distortions related to the B0 field was performed based on the acquired B0 field map.

At the subject level, the verbal N-back task was modelled as blocks using a general linear model (GLM) with four conditions: 0-back, 1-back, 2-back, and 3-back which were convolved with double-gamma hemodynamic response function with added temporal derivatives. These conditions were used to calculate a WM-load contrast which reflected a linear increase in activation from 0- to 3-back.

#### Pre-defined anatomical regions of interest

Based on our hypotheses, we constructed two volumes of interest (VOI) for the CCN and DMN, respectively, based on previous meta-analytic findings [37] and identical to our prior reports [12, 38]. The CCN VOI included the middle frontal gyrus, superior frontal gyrus, superior parietal lobule, parietal operculum cortex, supramarginal gyrus (anterior and posterior divisions), precuneus, angular gyrus, and postcentral gyrus. The DMN VOI consisted of the frontal medial cortex, hippocampal complex, and cingulate gyrus (posterior division). The regions included in both VOIs were generated using the FSLEyes Tool in FSL version 6 and defined by the Harvard-Oxford Cortical Structural Atlas [39], thresholded at 25%, based on a standard MNI template in the FSL package. We further constructed a region of interest (ROI) for the left dorsolateral prefrontal cortex (DLPFC) using a 10 mm radius sphere around MNI coordinates x = −44, y = 18, z = 22, based on previous verbal N-back WM reports [12, 40, 41].

#### fMRI group comparisons analysis

To examine group differences in WM-related neuronal response within the hypothesized left DLPFC ROI and CCN and DMN VOIs, respectively, we performed group-level analyses in FEAT with FMRIB’s Local Analysis of Mixed Effects (FLAME) as estimation method [42]. The model included the contrast of interest from the first-level analysis. The significance level for clusters was set at *p* < 0.05, corrected for multiple comparisons with a cluster-forming threshold of *Z* = 3.1 (*p* < 0.001). We performed an additional whole brain analysis using the same threshold as above to investigate any additional unpredicted group difference in neuronal activity. Mean percentage BOLD signal change was extracted from clusters showing significant differences between groups in WM-related activity for subsequent multiple linear regression analyses and illustrative purposes.

### Statistical analysis of neuronal response, cognition, and functioning associations in patients

To aid interpretation of identified neurocircuitry activity differences in patients vs. HC, we investigated associations between the extracted BOLD response and outside-scanner cognition or daily functioning using multiple regression analyses within the patient sample. We performed separate regression models with each of the identified clusters showing group differences as a predictor and each of the following outside-scanner behavioral variable as outcome: (1) ‘global cognition composite’ scores, (2) ‘working memory and executive functioning’ scores (average of the two domain composites most similar to the WM fMRI task), or (3) FAST total scores. All models were further adjusted for age, sex, IQ, and mood symptoms (HDRS-17 and YMRS scores). Due to multiple comparisons, the alpha level was Bonferroni-adjusted (alpha 0.05/number of comparisons).

### fMRI whole brain and behavior associations analysis

For our second aim to explore brain regions showing significant associations between WM-related neuronal response and cognitive performance and daily functioning, we set up a whole brain GLM in FEAT across the whole sample, using the contrast from the first-level analysis and the same statistical threshold as in the group comparisons analyses above. The GLM included a combined ‘working memory and executive functioning score’ (average of the two domain composites), the ‘global cognition’ score, and ‘FAST total’ scores as covariates in addition to group factors. To adjust for the effects of demographics and clinical variables on the relation between neuronal activity and cognition and functioning, we subsequently set up a similar GLM in FEAT, adding age, sex, IQ, HDRS-17 and YMRS scores as covariates in addition to the group, cognitive, and functioning factors. Variables were centered around their respective group means through demeaning. Finally, to explore if identified brain-behavior correlations significantly differed between patients vs. HC, we performed an additional GLM in FEAT with group-by-covariate interactions.

Following identification of brain regions showing correlations between neuronal response and cognitive and/or functioning scores, we conducted a conjunction analysis with cluster-level inference [43], assessing overlapping brain regions showing both group differences and correlations with behavioral scores. For the conjunction analysis, the significance level for clusters was set at 0.05 with a *Z*-threshold of 2.3.

## Results

### Sample characteristics

Table 1 displays the sample characteristics for patient and HC groups. The patient group was comparable to the HC group on sex, age, and IQ (*ps* ≥ 0.12), although patients had fewer years of education (patients: median = 14.46, IQR = 2.95; HC: median = 16, IQR = 2.09, *p* < 0.001). As expected, patients presented with more subsyndromal mood symptoms compared to HC (*ps* ≤ 0.001), although patients were in full or partial remission at the time of assessment (patients: HDRS-17: median = 4.0, IQR = 6.0; YMRS: median = 1.0, IQR = 3.0). Patients with mood disorders showed poorer cognitive performance compared to HC across all cognitive domain composites, also for the ‘global cognition’ score (*ps* < 0.001). Patients further had poorer daily functioning compared to HC (*p* < 0.001).

**Table 1:** Demographics, clinical, cognitive, and daily functioning characteristics in patients with mood disorders compared to healthy controls.

	Patients	Healthy controls	*t*/*U*/*χ*2	*p*-value
Demographics				
N	213	60		
Diagnosis, *n* (BD/MDD)	189/24			
Sex, *n* (%female)	140 (67%)	37 (62%)	0.34	0.56
Age, median (IQR)	32.00 (16.0)	28.50 (12.00)	5542.00	0.12
Years of education, mean (SD)	14.46 (2.95)	16.00 (2.09)	4.63	<0.001
Verbal IQ, mean (SD)	111.80 (5.54)	112.92 (4.85)	1.53	0.13
Clinical characteristics and functioning				
HDRS, median (IQR)	4.0 (6.0)	1.0 (2.0)	2181.00	<0.001
YMRS, median (IQR)	1.0 (3.0)	0.0 (0.0)	3985.00	<0.001
FAST, median (IQR)	19.0 (19.0)	0.0 (2.0)	325.00	<0.001
Antidepressants, *n* (%)	60 (28%)			
Antipsychotics, *n* (%)	68 (32%)			
Anticonvulsants, *n* (%)	84 (39%)			
Lithium, *n* (%)	92 (43%)			
Cognitive composite scores				
Global cognition, median (IQR)	−0.71 (0.88)	−0.01 (0.64)	10403.00	<0.001
Attention, median (IQR)	−0.62 (1.16)	−0.03 (0.69)	9586.00	<0.001
Psychomotor speed, median (IQR)	−1.03 (0.97)	0.00 (1.36)	9858.00	<0.001
Working memory, median (IQR)	−0.70 (1.23)	0.10 (0.93)	9664.50	<0.001
Verbal fluency and executive functioning, median (IQR)	−0.41 (0.68)	−0.16 (1.04)	8223.00	<0.001
Verbal learning and memory, median (IQR)	−0.80 (1.65)	0.25 (1.21)	9158.00	<0.001

*BD* bipolar disorder, *FAST* functioning assessment short test, *HDRS* hamilton depression rating scale – 17 Items, *IQR* interquartile range, *MDD* major depressive disorder, *SD* standard deviation, *YMRS* young mania rating scale.

### fMRI results

#### Group comparisons of neural response during working memory

**Fig. 1:**
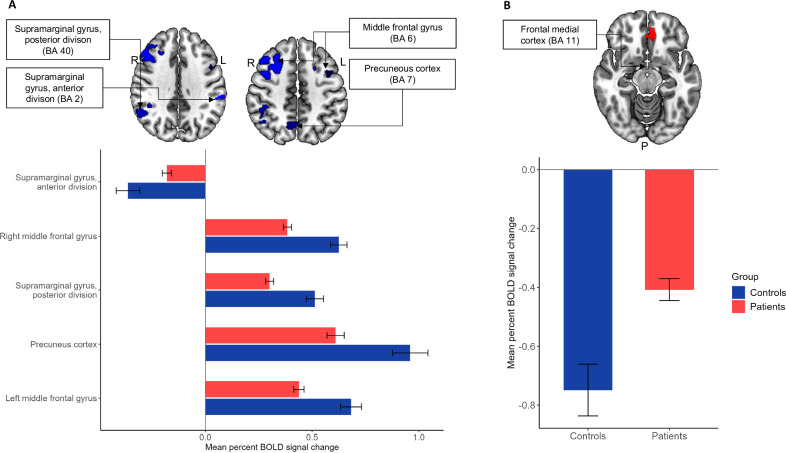
Brain regions showing statistically significant group differences in neural activity during working memory across the cognitive control network and default mode network in patients with mood disorders compared to healthy controls. **A**. Clusters in the cognitive control network showing aberrant activation in patients vs. controls. Patients showed hypo-activity in the left and right middle frontal gyrus, left supramarginal gyrus, posterior division, and precuneus cortex compared to controls. Patients further exhibited hyper-activity within the left supramarginal gyrus, anterior division. **B**. Clusters in the default mode network showing aberrant activation in patients vs. controls. Patients showed hyper-activation within the frontal medial cortex compared to controls. BA broadmann area, BOLD blood oxygen level dependent.

Table 2 lists clusters showing significant group differences in WM-related brain activation between patients and HC. Patients with mood disorders exhibited task-related hypo-activity compared to HC within the following regions of the CCN VOI: left and right middle frontal gyrus (MFG), right supramarginal gyrus, posterior division, and precuneus cortex (Fig. 1a). In contrast, patients displayed increased activity within the left supramarginal gyrus, anterior division of the CCN compared to HC (Fig. 1a). Within the DMN VOI, patients showed WM-related hyper-activity in the frontal medial cortex compared to HC (Fig. 1b). Within the left DLPFC ROI, patients further exhibited decreased activity during WM compared to HC (Table 2). These findings prevailed in a posthoc sensitivity analysis only comprising the BD patients vs. HC, suggesting that WM-related activity did not differ between BD and MDD patients (data not shown).

**Table 2:** Group differences in working memory-related neuronal response between patients with mood disorders and healthy controls.

		MNI					
Search area	BA	*x*	*y*	*z*	No. of voxels	Peak *Z*-value	*p*-value
CCN VOI							
Patients > controls							
L supramarginal gyrus, anterior division	2	−64	−32	28	129	3.97	0.007
Patients<controls							
L middle frontal gyrus	6	−42	8	44	175	4.24	0.002
R middle frontal gyrus	6	44	6	50	2342	5.52	<0.001
R supramarginal gyrus, posterior division	40	44	−44	42	439	4.70	<0.001
Precuneus cortex	7	10	−62	46	264	4.52	0.0002
DMN VOI							
Patients > controls							
Frontal medial cortex	11	−8	38	−12	119	3.95	0.002
Left DLPFC ROI							
Patients < controls							
L DLPFC	44	−50	12	24	38	4.04	0.003

*BA* brodmann area, *CCN* cognitive control network, *DLPFC* dorsolateral prefrontal cortex, *DMN* default mode network, *L* left, *MNI* montreal neurological institute, *R* right, *ROI* region of interest, *VOI* volume of interest.

Finally, the whole brain analysis showed that patients with mood disorders also exhibited decreased WM-related activity across additional frontal regions outside the CCN VOI, including the dorsal PFC, frontal pole, and frontal orbital cortex (see Supplementary Table S2).

### Associations between neuronal response and cognition and daily functioning in patients

One patient was missing a ‘global cognition composite’ score and was therefore excluded from the regression analyses, yielding a subsample of 212 patients. Supplementary Table S3 lists the *F-*statistics, adjusted *R*^2^, β-coefficients and *p*-values for the multiple regression analyses of BOLD response, cognition, and daily functioning within patients. The Bonferroni correction yielded an alpha level of 0.05/21 = 0.002 based on the seven identified clusters showing significant group differences above and the three behavioral outcomes (‘working memory and executive functioning’, ‘global cognition’, and FAST total scores, respectively). All regression models were statistically significant (*ps* < 0.001). Specifically, WM-related activity in the left MFG of the CCN was positively associated with ‘global cognition’ (β = 0.41, *p* = 0.001) and ‘working memory and executive functioning’ (β = 0.34, *p* = 0.002) in patients, after adjusting for age, sex, IQ, and HDRS-17 and YMS scores. There was also a trend towards a positive association between BOLD response in the right MFG and ‘global cognition’ (β = 0.43, *p* = 0.006). Brain activity in the remaining clusters were not significantly associated with cognition or daily functioning in patients after multiple comparisons correction (*ps* ≥ 0.012).

### Exploratory analyses of brain regions showing associations between BOLD activity and cognition and functioning

**Fig. 2:**
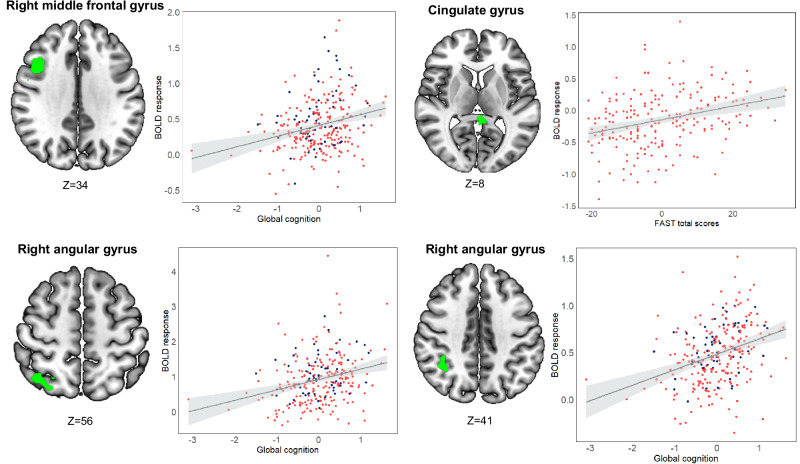
Brain areas showing significant associations between global cognition or daily functioning and neuronal response during working memory in patients with mood disorders (red) and healthy controls (blue). The patient data is only shown for the correlations among BOLD response and functioning, as there was minimal variability in the FAST scores for the healthy controls. A higher FAST score reflects poorer functional capacity. BOLD blood oxygen level dependent, FAST functional assessment short test.

The patient missing a ‘global cognition composite’ score was also excluded from the exploratory whole brain GLMs with behavioral data, resulting in a subsample of 212 patients and 60 HC. The whole brain GLMs complemented the findings from the hypothesis-driven analyses above, identifying three clusters of the CCN showing significant correlations between WM-related activity and ‘global cognition’, as well as one cluster in the DMN associated with daily functioning (Table 3; Fig. 2). Specifically, global cognition was positively associated with BOLD response within the right MFG (*p* = 0.0005) and two clusters within the right angular gyrus (*ps* ≤ 0.034) (Table 3). When adjusting for age, sex, IQ, and mood symptoms, the right MFG cluster remained significant (*p* = 0.03). Further, daily functioning (i.e., FAST total scores) was positively associated with WM-related response within the cingulate gyrus, posterior division in the DMN (*p* = 0.0068; Table 3), which also prevailed after covarying for age, sex, IQ, and mood symptoms (*p* = 0.04). Since the FAST scale is designed for clinical populations, the majority of HCs received a score of 0, leading to minimal variability in the HC data. We therefore conducted a sensitivity analysis in patients only, which confirmed the positive correlation between cingulate gyrus activity and FAST scores (*p* = 0.039; shown in Fig. 2). The GLMs revealed no clusters showing significant associations between ‘working memory and executive functioning’ and WM-related BOLD response. The additional GLM with group-by-covariate interactions showed no significant interactions, suggesting that the observed correlations between BOLD response and cognition and functioning were similar across patients and HC.

**Table 3:** Clusters showing significant associations between working memory-related neuronal activity and global cognition and daily functioning across patients with mood disorders and healthy controls.

		MNI					
Search area	BA	*x*	*y*	*z*	No. of voxels	Peak *Z*-value	*p*-value
Global cognition composite							
R middle frontal gyrus	44	42	12	34	277	4.4	0.0005^a^
R angular gyrus	7	42	−60	54	138	3.8	0.025
R angular gyrus	40	36	−54	40	128	4.13	0.034
FAST total scores							
Cingulate gyrus, posterior division	26	−4	−38	8	181	4.38	0.0068^a^

*BA* brodmann area, *FAST* functioning assessment short test, *HDRS* hamilton depression rating scale - 17 items, *L* left, *MNI* montreal neurological institute, *R* right, *YMRS* young mania rating scale.

^a^Remained statistically significant after adjusting for age, sex, IQ, HDRS-17 and YMRS scores.

The conjunction analysis assessing brain regions showing both WM-related differential activity in patients vs. HC and a correlation with behavior identified two overlapping brain clusters: the right MFG (*p* = 0.048) and the right angular gyrus (*p* = 0.025), which showed hypo-activity in patients vs. HC in addition to positive correlations with global cognition across the sample, respectively.

## Discussion

This is the largest fMRI study to date to examine brain activity underlying cognition and daily life functioning in full or partially remitted patients with mood disorders. Patients showed aberrant (mainly hypo-) activation within frontal and parietal nodes of the CCN during a verbal WM N-back task, which further correlated with the observed impairment across comprehensive measures of outside-scanner working memory and executive functioning and global cognition in patients. We also found that patients displayed hyper-activity within the frontal medial cortex node of the DMN compared to controls, but this was not correlated with outside-scanner cognition or functioning. Finally, we demonstrated that WM-related activity within the right MFG and angular gyrus of the CCN were positively associated with global cognitive performance across the whole sample, whereas greater activation within the posterior cingulate gyrus, a key hub of the DMN, was associated with more functional disability.

Our finding of patients showing aberrant DLPFC and CCN hypo-activity related to cognitive impairment is in line with prior reports in mood disorders [44–46], also across different mood states [47]. The CCN is involved in explicit cognitive control processes, including response initiation and planning processes [48]. The observed hypo-activity within this network in patients vs. HC could hence reflect that patients are not able to sufficiently recruit the cortical resources required during cognitively demanding tasks, which leads to poorer performance [9]. In particular, we identified three brain clusters in the frontal and parietal nodes of the CCN showing positive correlations with global cognition across the whole sample, including the right MFG and right angular gyrus. This suggests that WM-related activation within this network plays a key role in global cognitive performance across domains. Hence, our overlapping results from the analyses, as confirmed by the conjunction analysis, add to compiling evidence showing that aberrant *hypo*-activity within the CCN may underlie *detectable* cognitive impairment in patients with mood disorders [9]. Notably, this observed characterization of CCN hypo-activity underpinning cognitive impairment does not seem to be specific to mood disorders only, but extends to other neuropsychiatric conditions such as schizophrenia [49], obsessive compulsive disorder [50], and attention deficit hyperactivity disorder [51]. This implies a *trans*diagnostic neurocircuitry dysfunction underlying wide-spread cognitive impairment.

We also confirm our hypothesis that patients show hyper-activity within the DMN, specifically the frontal medial cortex, compared to HC. This is in line with prior reports on aberrant increased DMN activity in patients with mood disorders [11, 46, 52]. The DMN is involved in self-referential cognitive processes such as autobiographical recall, and it is generally disengaged during explicit cognitive performance in healthy individuals [53]. Accordingly, the DMN has also been related to cognitive performance in terms of attention lapses and distractive mind-wandering [54]. Nevertheless, we did not observe any association between WM-related DMN activity and cognition in the present study, indicating that (dis)engagement of the DMN was not directly related to participants’ outside-scanner cognitive abilities. Instead, we identified a cluster in the posterior cingulate gyrus, a key hub in the DMN, showing a correlation between higher task-related activity and poorer daily life functioning. It has been suggested that aberrant DMN hyper-activity may be related to patients’ subsyndromal symptoms, which may evoke more self-referential and ruminative thoughts that negatively impact functioning [11, 52]. Interestingly, we found that the association between DMN activity and daily functioning prevailed after adjusting for subsyndromal depressive and (hypo)manic symptoms, suggesting that patients’ failure to disengage the DMN may constitute a neurocircuitry *trait* in mood disorders independent of current mood status. Based on this, the observed DMN hyper-activity in patients may instead reflect a failure to suppress irrelevant social cognitive processes, such as spontaneous autobiographical memories and self-reflection, during cognitively demanding challenges such as the N-back task [55]. Indeed, these DMN-related cognitive processes may have a considerable impact on patients’ daily functioning as captured by the FAST scale, since everyday life often lacks clear structure and is frequently marked by various distractions. This may also explain the absence of correlation between DMN activity and cognition in this study, as the cognitive testing occurred in a quiet and structured setting that reduces the impact of spontaneous DMN-related attention lapses and self-referential thinking on patients’ cognitive performance. Nevertheless, our findings contrast to prior studies investigating the relationship between brain activity and functioning in mood disorders, which found hypo-activity in the PFC and CCN during memory encoding and WM tasks to be related to poor functional capacity [16–18, 56], although the sample sizes were considerably smaller in these studies (*n* = 20–91 subjects) compared to the present report.

The findings have implications for treatment strategies that aim to improve cognition and functioning in mood disorders. Our results point to aberrant DLPFC and CCN hypo-activity as robust neurocircuitry correlates of cognitive impairment in mood disorders. Engagement of aberrant neurocircuitry activity in these regions may thus reflect key targets for pro-cognitive interventions. Indeed, this is consistent with emerging findings from mechanistically diverse pro-cognitive intervention trials assessing treatment-related neuronal changes associated with cognitive improvement in mood disorders [19]. In these trials, treatment-related cognitive improvement has been accompanied by a normalization of patients’ aberrant task-related activity within the dPFC and parietal nodes of the CCN in response to both strategy-based behavioral interventions like cognitive remediation [57] and pharmacological treatments, such as erythropoietin [58, 59] and vortioxetine [40]. Conversely, a negative cognitive remediation trial in BD found *no* change in dPFC engagement following an ineffective treatment [60], which underscores both the sensitivity and specificity of the observed link between dPFC activity changes and cognitive enhancement. In particular, cognitive impairment and associated dPFC and CCN hypo-activity is likely to arise from disruptions in neuroplasticity, including reduced neurogenesis and excessive pruning, due to low-grade inflammation, oxidative stress, and low levels of neurotrophins [61]. Thus, upregulation of neuroplasticity with different treatments with distinct underlying mechanisms (e.g., erythropoietin or intensive, ‘plasticity-based’ cognitive remediation) may result in similar treatment-related dPFC and CCN activity changes detectable at a systems level by fMRI. As such, future pro-cognitive treatment trials should include WM fMRI tasks to investigate treatment-related target engagement within these key neurocircuitries, in line with recent expert recommendations [19]. Moreover, our finding of DMN hyper-activity associated with functional disability suggests a putative neurocircuitry biomarker that can be utilized in future research to evaluate efficacy of interventions that aim to improve functioning in mood disorders. As such, mental health interventions that have previously shown to modulate DMN response - including meditation [62] and psilocybin-assisted mindfulness training [63] - could lead to functional improvements in patients. Notably, these effects may result from reductions in self-reflection and rumination [52], enabling a reorientation in attention towards the external world and thereby engagement in new behavioral patterns that may enhance daily life functioning. It would thus also be interesting to include fMRI in future intervention studies that aim to improve functioning in mood disorders to explore treatment-related DMN target engagement.

Strengths of the present fMRI study include that it is the largest of its kind to date, involving a sample of 213 full or partially remitted patients with mood disorders and 60 HC, which contrasts to prior imaging studies with generally *n* < 50 subjects. Another strength was the associations between WM-related brain activity and comprehensive outside-scanner measures of wide-spread cognitive performance and objective daily functioning, which highlights the *functional* relevance of the observed neurocircuitry alterations [64]. Finally, we applied an integrative approach, combining both hypothesis-driven and exploratory methods, that robustly identified neurocircuitry dysfunctions in networks relevant for cognition and functioning both in patients and across the whole sample. A limitation was that we used previously published data from a majority of the participants (*n* = 233 participants) included in the present cohort [12, 41]. Nevertheless, this study differs from our prior reports, which focused on patients with BD only [12] or on patients with only objectively verified cognitive impairment [41], which may limit generalizability of findings due to the cognitive heterogeneity in patients with mood disorders [65]. Further, the present study aimed to utilize a combination of hypothesis-driven and exploratory approaches to investigate transdiagnostic neuronal correlates of daily functioning and cognition in mood disorders, also in contrast with our previous reports. The present study also included samples of patients with newly diagnosed disorder as well as patients with longer illness durations, which potentially may have influenced findings. Another limitation is that patients took different medication types and doses, which could have affected their neuronal response. However, medication status seems to have relatively limited confounding effects on neuroimaging findings [66]. Finally, the cross-sectional study design limited any causal inferences between brain activity and cognition and functioning.

In conclusion, the present study adds to compiling evidence pointing to aberrant CCN hypo-activity, possibly combined with DMN hyper-activity, as key neuronal correlates of cognitive impairment and functional disability in patients with mood disorders. Future trials that aim to improve cognition and daily life functioning in mood disorders should implement WM-related fMRI tasks to examine target engagement within those brain networks and associated clinical efficacy.

## Supplementary information

Supplementary material

## Data Availability

The data used and analyzed in the current study are available from the corresponding author upon reasonable request.
