# Supplementary material for: Network-wide aberrancies in neuronal activity during working memory in a large cohort of patients with mood disorders: associations with cognitive impairment and functional disability
Source: Mol Psychiatry. 2025 Jun 17;30(10):4836–44. doi: 10.1038/s41380-025-03078-x (PMC12436155; doi:10.1038/s41380-025-03078-x)
Supplement: Supplementary file 1 — Supplementary material [file 41380_2025_3078_MOESM1_ESM.pdf]

Supplementary material

Table S1. Cognitive tests grouped within each cognitive composite score

**Attention**	**Psychomotor speed**	**Working memory**	**Verbal fluency and executive function**	**Verbal learning and memory**
TMT-A	RBANS Coding	WAIS Letter-Number Sequencing	TMT-B	RAVLT Trial I-V correct
RBANS Digit Span Forward	SCIP-PST	SWM between errors	Verbal fluency S	RAVLT Trial IV correct
RVP Accuracy		SWM strategy	Verbal fluency D	RAVLT Delayed recall
RVP latency		SCIP-WMT	OTS mean choices to correct	RAVLT Recognition
			OTS mean latency	SCIP-VLT Immediate
			SCIP-VFT	SCIP-VLT Delayed

Abbreviations: OTS=One Touch Stockings of Cambridge; PST=Psychomotor Speed Test; RBANS=Repeatable Battery for the Assessment of Neuropsychological Status; RAVLT=Rey Auditory Verbal Learning Test; RVP=Rapid Visual Processing; SCIP=Screening for Cognitive Impairment in Psychiatry; SWM=Spatial Working Memory; TMT= Trail Making Test; VFT=Verbal Fluency Test; VLT=Verbal Learning Test; WAIS=Wechsler Adult Intelligence Scale; WMT=Working Memory Test.

Table S2. Group differences in neuronal response between patients with mood disorders and healthy controls during working memory at the whole brain level

Search area	BA	MNI *x*	*y*	*z*	No. of voxels	Peak *Z*-value	Peak *p*-value
**Whole brain**							
Patients > controls							
Frontal medial cortex	11	-10	32	-12	387	4.25	<.001
L supramarginal gyrus, anterior division	2	-64	-32	28	150	3.97	0.018
Patients < controls							
L middle frontal gyrus	6	-42	8	44	267	4.24	<.001
R middle frontal gyrus	6	44	6	50	3920	5.52	<.001
L frontal pole	11	-30	54	4	215	4.85	0.003
R Frontal pole	10	36	62	-2	190	4.17	0.006
R dorsal prefrontal cortex	0	36	64	20	310	5.06	<.001
R frontal orbital cortex	47	38	24	-4	269	4.94	<.001
L frontal operculum cortex	48	-36	22	4	388	4.88	<.001
R supramarginal gyrus, posterior division	40	44	-44	42	1117	4.7	<.001
Precuneous cortex	0	10	-62	46	451	4.52	<.001
R caudate	0	16	6	20	158	5.18	0.014
Cerebellum	0	-4	-64	-12	597	4.3	<.001
R thalamus	0	6	-4	-2	486	4.76	<.001
Brain stem	0	8	-28	-14	291	4.69	<.001

Abbreviations: BA=Brodmann area; L=left; MNI=Montreal Neurological Institute; R=right.

Table S3. Associations between extracted neuronal response and cognitive performance and daily functioning in patients with mood disorders.

		Model			Predictor	
**Predictor**	**Outcome**	***F***	**Adj. R^2**	***p*-value**	**β**	***p*-value**
Left DLPFC	WM+EF	12.92	0.26	**<.001***	0.21	.012
	Global cognition	9.49	0.20	**<.001***	0.24	.015
	FAST	6.65	0.14	**<.001***	-1.63	.40
Right middle frontal gyrus (CCN)	WM+EF	12.62	0.25	**<.001***	0.30	.026
	Global cognition	9.87	0.20	**<.001***	0.43	.006
	FAST	6.51	0.14	**<.001***	-0.35	.91
Left middle frontal gyrus (CCN)	WM+EF	13.76	0.27	**<.001***	0.34	**.002***
	Global cognition	10.43	0.21	**<.001***	0.41	**.001***
	FAST	6.53	0.14	**<.001***	-0.90	.72
Precuneous cortex (CCN)	WM+EF	12.47	0.25	**<.001***	0.14	.038
	Global cognition	9.44	0.20	**<.001***	0.18	.017
	FAST	6.55	0.14	**<.001***	0.66	.66
Supramarginal gyrus (posterior division) (CCN)	WM+EF	12.16	0.24	**<.001***	0.24	.083
	Global cognition	8.99	0.19	**<.001***	0.31	.058
	FAST	6.55	0.14	**<.001***	-1.38	.66
Supramarginal gyrus (anterior division) (CCN)	WM+EF	12.34	0.25	**<.001***	-0.24	.051
	Global cognition	9.03	0.19	**<.001***	-0.28	.051
	FAST	6.54	.014	**<.001***	1.05	.70
Frontal medial cortex (DMN)	WM+EF	12.35	0.25	**<.001***	-0.13	.051
	Global cognition	9.02	0.19	**<.001***	-0.15	.053
	FAST	6.51	0.14	**<.001***	-0.17	.91

*Significant at corrected alpha level of 0.002. Adjusted for age, sex, IQ, and HDRS and YMRS scores.

Abbreviations: Adj.=Adjusted; BD=Bipolar disorder; CCN=Cognitive Control Network; DLPFC=Dorsolateral prefrontal cortex; DMN=Default mode network; FAST=Functional Assessment Short Test; HDRS: Hamilton Depression Rating Scale; UD=Unipolar depression; YMRS=Young Mania Rating Scale.
